# Unsuspected drug shortage is impacting the German HIV PrEP supply - results of a cross-sectional survey show: majority of PrEP users had to stop or switch to on-demand PrEP and higher unmet PrEP demand among women, diverse individuals, and those in rural or small-town areas

**DOI:** 10.1186/s12879-025-12086-9

**Published:** 2025-11-13

**Authors:** Daniel Schmidt, Göran Kirchner, Konstantinos Voulgaris, Martin Friebe, Viviane Bremer, Barbara Bartmeyer

**Affiliations:** Department of Infectious Disease Epidemiology, Robert Koch Institute, Berlin, Germany

**Keywords:** HIV pre-exposure prophylaxis (PrEP), Germany, Drug supply shortage, Unmet PrEP demand

## Abstract

**Background:**

To assess the implications of HIV pre-exposure prophylaxis (PrEP) shortages, we conducted a survey among PrEP users and individuals interested in PrEP. The survey explored coping strategies, HIV prevention and sexual behavior and stress levels due to the drug shortages. Furthermore, barriers to PrEP access among people with unmet PrEP demand were investigated.

**Methods:**

An anonymous online survey was conducted from 28.01.-27.02.2024. Recruitment occurred via social media, professional societies, network channels, and flyers at HIV specialists. Factors associated with high stress levels and unmet PrEP demand were investigated using multivariable logistic regression.

**Results:**

Among 926 PrEP users, most were male (98%), aged 31–45 (54%), identified as men (97%), lived in large cities (54%), and used PrEP daily (78%). Due to the shortage, 63% paused or switched to on-demand use. Most intended to resume PrEP once available (65%), followed by relying on condoms (41%) or reducing sexual partners (39%). High stress level was reported by 78%. Predictors of higher stress levels included feeling less protected against HIV and having more than 11 sexual partners; using on-demand PrEP with fewer than 20 monthly pills was significantly less likely. Unmet PrEP demand was more common among female and gender-diverse individuals and less common among those living in large cities.

**Conclusions:**

Involuntary changes in PrEP use may have hindered users’ ability to adapt sexual behavior, increasing HIV risks. PrEP users most at risk and most reliant on PrEP experienced greater psychological burden. Expanding PrEP education and outreach alongside with prescriber capacity is essential to ensure PrEP continuity and meet unmet PrEP demand. Individualized counseling could help to determine actual PrEP need and benefits.

**Clinical trial number:**

Not applicable.

**Supplementary Information:**

The online version contains supplementary material available at 10.1186/s12879-025-12086-9.

## Background

Since September 2019, people with statutory health insurance (SHI) who are at substantial risk of HIV infection have been entitled to coverage of HIV pre-exposure prophylaxis (PrEP). The coverage of PrEP includes counseling, the continuous supply of medication and examinations, such as HIV and risk-based STI testing [1]. The Robert Koch Institute (RKI) was commissioned by the German Federal Ministry of Health (BMG) to scientifically monitor and evaluate the introduction of PrEP as a new service of the SHI which was carried out in the PrEP evaluation project (EvE-PrEP) [2]. The results of the EvE-PrEP showed that PrEP is highly effective, but that there were gaps in provision and potential for improvement [3]. Based on the results of the EvE-PrEP, the RKI planned to establish a continuous monitoring of HIV-PrEP provision in Germany from 2022 as part of the project “Surveillance of HIV PrEP within the statutory health insurance system in Germany (PrEP-Surv)”. This project ended in 2024 due to a lack of further funding [4].

In October 2023, first reports of tenofovir disoproxil/emtricitabine (TDF/FTC) supply shortage were communicated. This antiretroviral drug combination is used for HIV therapy as well as for PrEP. The shortage likely resulted from multiple factors, including production problems, supply chain disruptions, and structural aspects of Germany’s procurement system, with further contributing causes possible. After initial assurances of the manufacturers that the situation was stable and that the supply shortage would eventually disappear, the situation deteriorated significantly over time. As a result, various players in the HIV sector appealed to the responsible authorities which lead to corresponding exchanges and joint efforts [5, 6]. With the result that on January 25, 2024, the official supply shortage was announced in accordance with § 79 (5) of the German Medicines Act, which opened up further legal opportunities in the procurement and provision of medicines. For example, pharmacies were allowed to dispense the more expensive brand-name medication. As a result of the combined efforts of community organizations, professional societies, activists and politics and the actions taken, the supply shortage eased increasingly from spring 2024 [7].

To assess the critical situation caused by the TDF/FTC supply shortage, an anonymous online survey was promptly planned and conducted as part of the PrEP-Surv project. The aim of the survey was to analyze the impact of the supply shortage on PrEP users. It focused on strategies for dealing with the shortage, including changes in PrEP sources, HIV prevention behavior, and sexual behavior. Furthermore, participants’ perceptions of HIV protection and stress were examined. Additionally, factors associated with unmet PrEP demand were analyzed.

## Methods

We conducted a cross-sectional study on the effects of TDF/FTC supply shortage on PrEP users. People with unmet PrEP demand were asked about the reasons for not using PrEP yet. The planning and implementation of the anonymous online survey took place from January 10–18, 2024. The survey was then conducted from January 28 to February 27, 2024 and was offered in German and English (see supplement for the English version of the questionnaire). The link to the online questionnaire was distributed via the prep.jetzt Facebook and WhatsApp groups. Additionally, the link was shared through different RKI social media channels, the Fast-Track City network Berlin, and the professional societies: German Association of Outpatient Physicians for Infectious Diseases and HIV Medicine (dagnä), German AIDS Society (DAIG) and German STI Society (DSTIG). Furthermore, dagnä HIV specialist centers were asked to display a flyer with a QR code in their centers. This approach aimed to reach a sufficiently broad population of PrEP users as well as individuals with PrEP demand. The survey was open to PrEP users and individuals with an interest in PrEP. No further eligibility criteria were applied, and participation was based on voluntary response and convenience sampling.

### Statistical analysis

The answers to categorical items were displayed as absolute numbers or proportions. The questionnaire contained no continuous variables.

Factors associated with high or very high levels of stress were investigated using multivariable logistic regression. Co-variates included age, PrEP intake mode, perception of HIV protection, number of different sexual partners (anal, vaginal) in the last six months and the residential area of participants by population size.

Factors associated with unmet PrEP demand were investigated using chi²-Test and multivariable logistic regression. Co-variates for the adjusted model included sex assigned at birth, age and the residential area of participants by population size.

Descriptive statistics and further analyses including graphs and figures were conducted using R version 4.1.3 and Microsoft Excel.

## Results

### Study participation

**Fig. 1:**
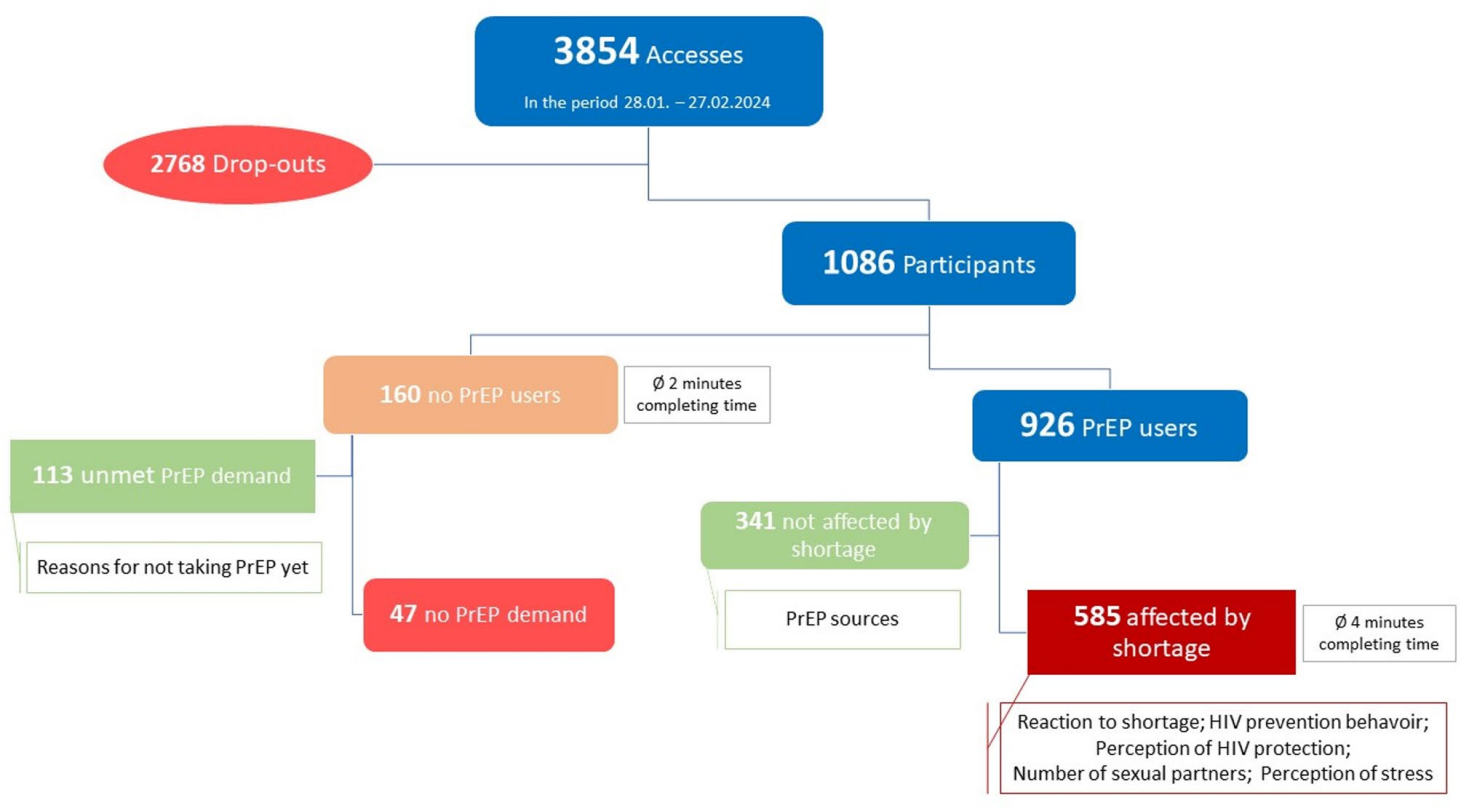
Accessed and completed questionnaires from the online survey on dealing with the supply TDF/FTC shortage

In the period 28.01. − 27.02.2024, the online questionnaire was accessed 3854 times. A total of 1086 participants completed the questionnaire in full, 926 of whom were PrEP users, including 585 people who stated that they were affected by the supply shortage. In addition, there were 160 people who did not use PrEP, 113 of whom reported an unmet PrEP demand (Fig. 1). Of all questionnaires, 1033 (95%) were completed in German and 55 (5%) in English. The average duration to complete the questionnaire was 3.8 min, 4.1 min for PrEP users and 2 min for non-PrEP users.

### Regional distribution of participants

**Fig. 2:**
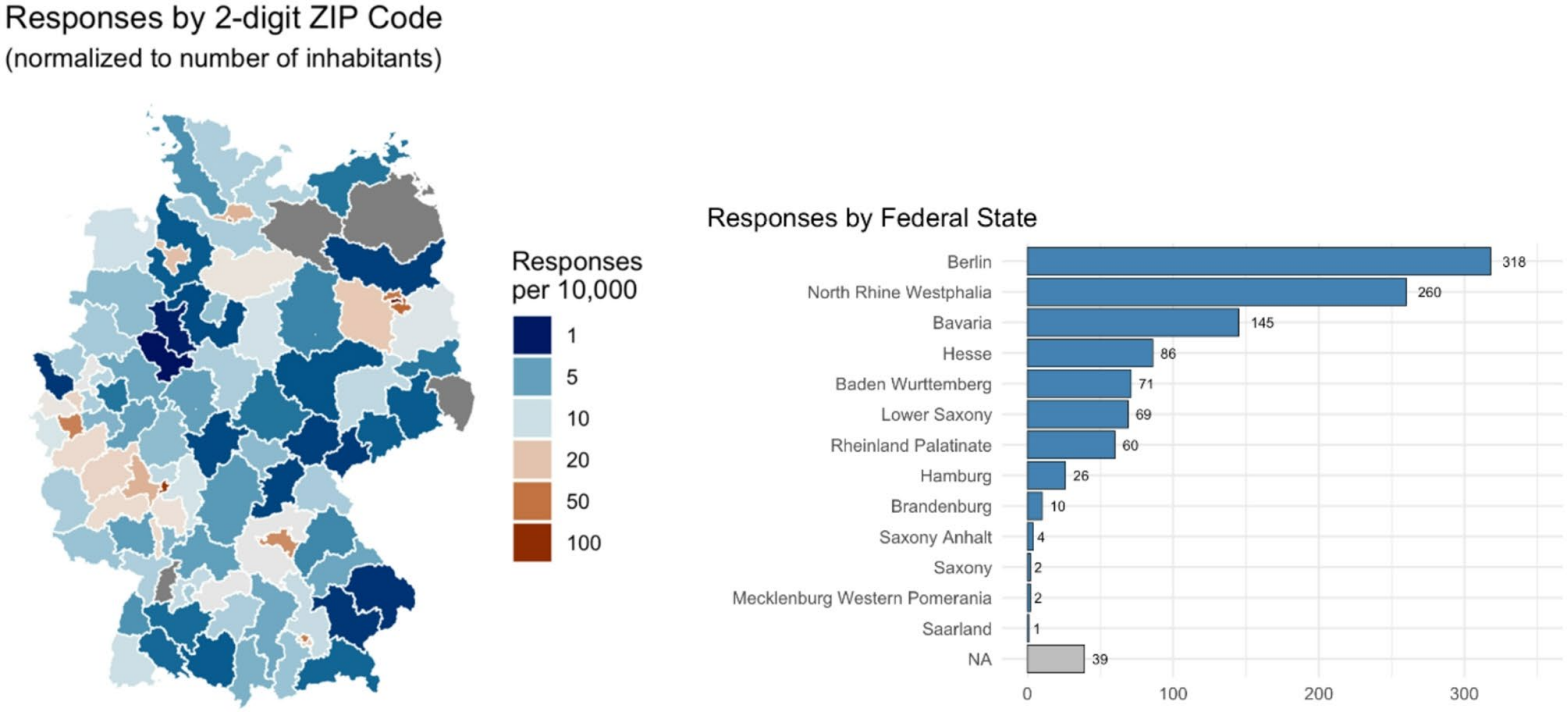
Responses by 2-digit ZIP code (normalized to number of inhabitants) zero values in grey & responses by federal state (*N* = 1,086)

The regional distribution of the 1086 participants with complete questionnaires is shown in Fig. 2. There is a concentration in certain cities and urban regions as well as at the federal state level in certain areas, which is consistent with data on PrEP use and the PrEP care landscape, in particular the number of HIV specialty centers [8].

**Table 1:** Characteristics of the study population for PrEP users and people with unmet PrEP demand

		PrEP users	%	People with unmet PrEP demand	%	Total
Total		926	100.0%	113	100.0%	1039
Sex	Male	906	97.8%	99	87.6%	1005
	Female	16	1.7%	10	8.8%	26
	Diverse	4	0.4%	4	3.5%	8
	No answer	0	0.0%	0	0.0%	0
Gender identity	Man	896	96.8%	98	86.7%	994
	Woman	13	1.4%	8	7.1%	21
	Non-binary	22	2.4%	7	6.2%	29
	Trans-Man	6	0.6%	4	3.5%	10
	Genderfluid	13	1.4%	4	3.5%	17
	Trans-Woman	4	0.4%	2	1.8%	6
	Agender	3	0.3%	2	1.8%	5
	Inter	2	0.2%	0	0.0%	2
	No answer	8	0.9%	4	3.5%	12
Age	16–20	6	0.6%	2	1.8%	8
	21–25	49	5.3%	16	14.2%	65
	26–30	107	11.6%	16	14.2%	123
	31–35	173	18.7%	18	15.9%	191
	36–40	170	18.4%	22	19.5%	192
	41–45	154	16.6%	14	12.4%	168
	46–50	110	11.9%	10	8.8%	120
	51–60	128	13.8%	13	11.5%	141
	> 60	29	3.1%	2	1.8%	31
	No answer	0	0.0%	0	0.0%	0
Residential area of participants by population size	Large city ( > = 500,000 inhabitants)	499	53.9%	47	41.6%	546
	Medium-sized city ( > = 200,000-<500,000 inhabitants)	112	12.1%	14	12.4%	126
	Small town or rural area (< 200,000 inhabitants)	290	31.3%	47	41.6%	337
	No answer	25	2.7%	5	4.4%	30
First PrEP start	Before 2017 (2010–2016)	41	4.4%			41
	2017	53	5.7%			53
	2018	100	10.8%			100
	January-August 2019	92	9.9%			92
	September 2019	54	5.8%			54
	2020	109	11.8%			109
	2021	114	12.3%			114
	2022	163	17.6%			163
	2023	184	19.9%			184
	Until January/February 2024	20	2.2%			20
	No answer	0	0.0%			0
PrEP use	daily	719	77.6%			719
	on-demand more than 20 pills/month	85	9.2%			85
	on-demand less than < 20 pills/month	122	13.2%			122
	No answer	0	0.0%			0
Affected by PrEP shortage	Yes	585	63.2%			585
	No	341	36.8%			341
	No answer	0	0.0%			0
Since when affected by PrEP shortage	Total of PrEP users affected	585	100.0%			585
	October 2023	18	3.1%			18
	November 2023	43	7.4%			43
	December 2023	130	22.2%			130
	January 2024	394	67.4%			394
	No answer	0	0.0%			0
Number of different sexual partners (anal or vaginal) of PrEP users in the last six months	> 20	154	26.3%			
	11–20	83	14.2%			
	6–10	137	23.4%			
	4–5	85	14.5%			
	2–3	80	13.7%			
	1	21	3.6%			
	0	10	1.7%			
	No answer	15	2.6%			
Perception of HIV protection	I feel very well protected	113	19.3%			
	I feel well protected	173	29.6%			
	I feel less well protected	196	33.5%			
	I do not feel protected	103	17.6%			

### Analysis of PrEP users and their strategies to deal with the supply shortage

**Fig. 3:**
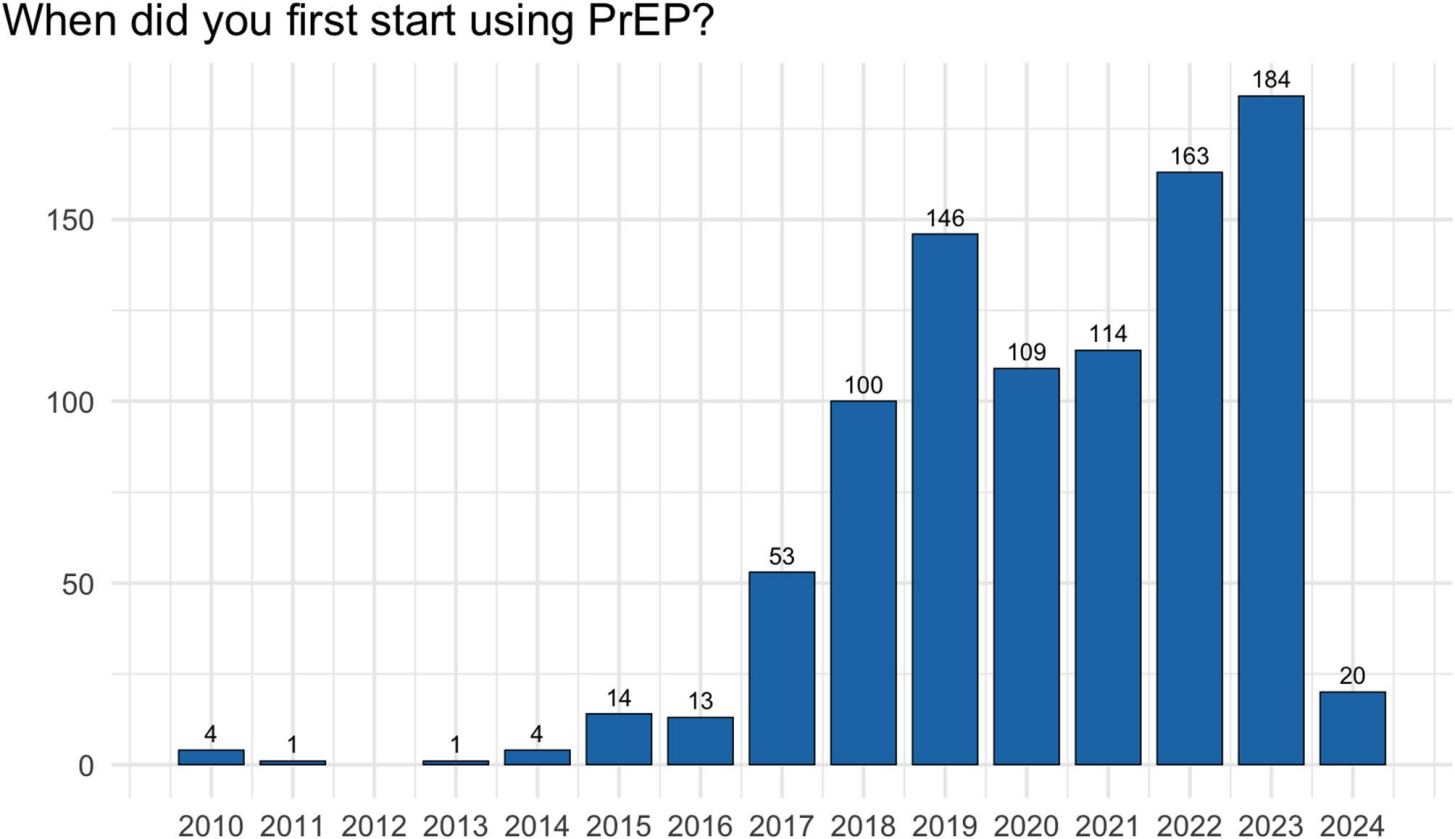
Information on the first PrEP start of PrEP users (*N* = 926) over time

The 926 PrEP users were asked about their first PrEP start and PrEP intake mode. Overall, 282 (30%) of PrEP users had already started PrEP before the introduction of PrEP as a statutory health insurance benefit in September 2019, while 644 (70%) had started in or after September 2019. The comparison over the time shows a lower number of PrEP starts in 2020 and 2021 compared to 2019, 2022 and 2023 (Fig. 3), which is probably related to changes in sexual behavior and barriers to access during the COVID-19 pandemic.

A total of 719 (78%) PrEP users reported continuous/daily PrEP, followed by 122 (13%) reporting PrEP on-demand with less than 20 pills per month and 85 (9%) reporting PrEP on-demand with more than 20 pills per month. This corresponds to 804 PrEP users (87%) reporting intake patterns indicating high PrEP pill coverage.

## PrEP supply sources

**Fig. 4:**
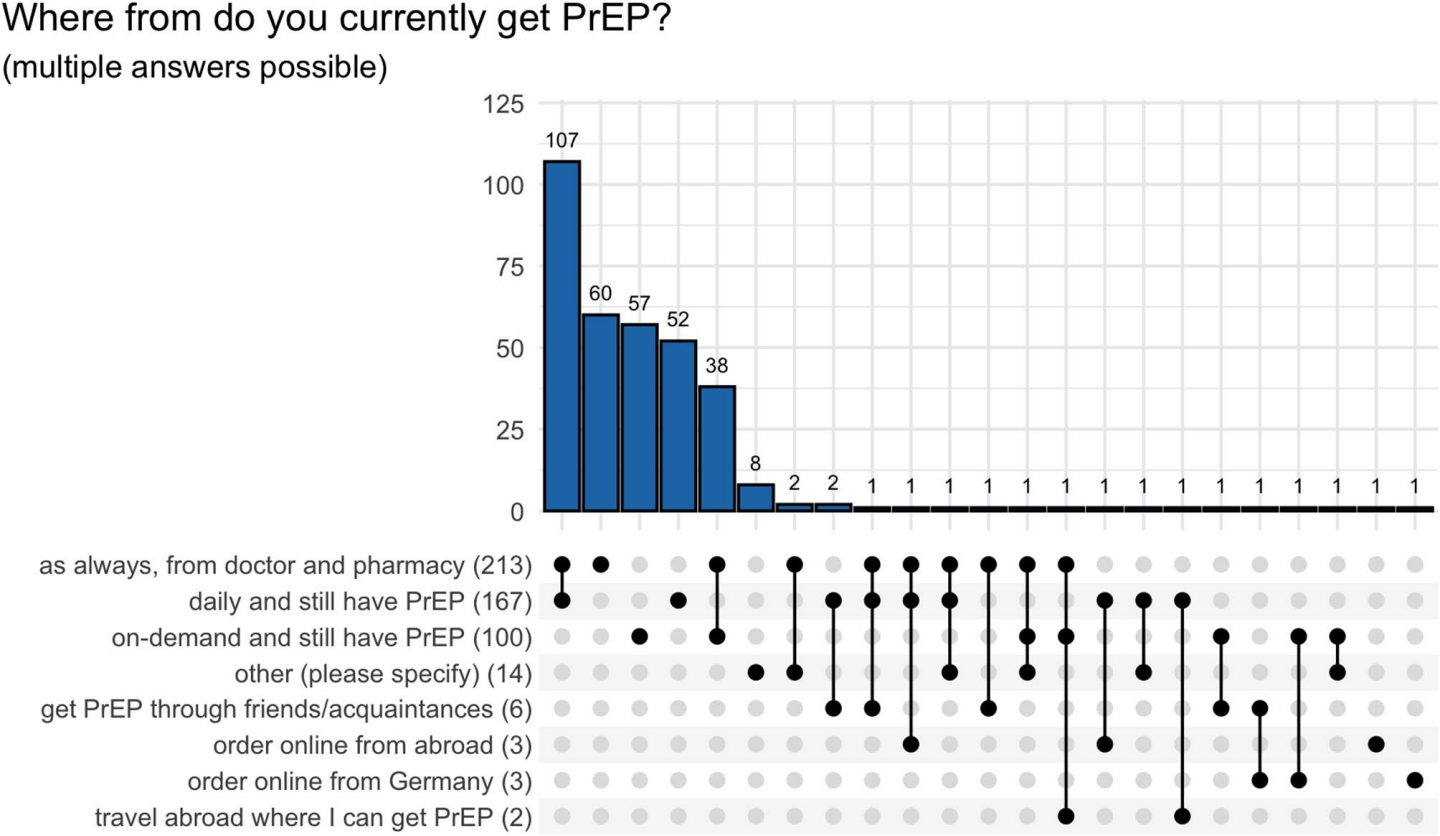
PrEP supply sources of PrEP users who stated that they were not affected by the supply shortage (*N* = 341), multiple answers possible (*N* = 508), number of answers for each row in parenthesis

PrEP users were asked to what extent they were affected by the current supply shortage, i.e. whether they had already had difficulties obtaining PrEP in the current situation. Of all 926 PrEP users, 585 (63%) PrEP users stated that they were affected by the supply shortage, while 341 (37%) PrEP users stated that they were not affected by the supply shortage. PrEP users who were not affected by the supply shortage mainly indicated the following sources of PrEP supply (multiple answers possible): “I get PrEP as usual from my doctor and pharmacy” (*N* = 213, 62%), “I currently take PrEP daily and still have PrEP” (*N* = 167, 49%),” I currently take PrEP on an occasional/on-demand basis and still have PrEP” (*N* = 100, 29%), see Fig. 4. We used an UpSet plot to illustrate the multiple answers given, with the rows corresponding to the answers chosen, and the columns to the combination of those choices (marked with a black dot and linked by a line). An UpSet plot effectively visualizes the combination of multiple answers [9]. As an example of how to interpret the UpSet plot: of the 213 participants who reported getting PrEP ‘as always, from doctor and pharmacy’ (i.e., the combination of all rows indicating this), 107 also indicated ‘daily and still have PrEP.’ In total, 167 participants reported ‘daily and still have PrEP,’ considering all combinations of answers for this row.

The PrEP users who stated that they had been affected by the supply shortage (*N* = 585) had mainly been affected since January 2024 (*N* = 394, 67%), see Table 1.

## Reaction to the supply shortage

**Fig. 5:**
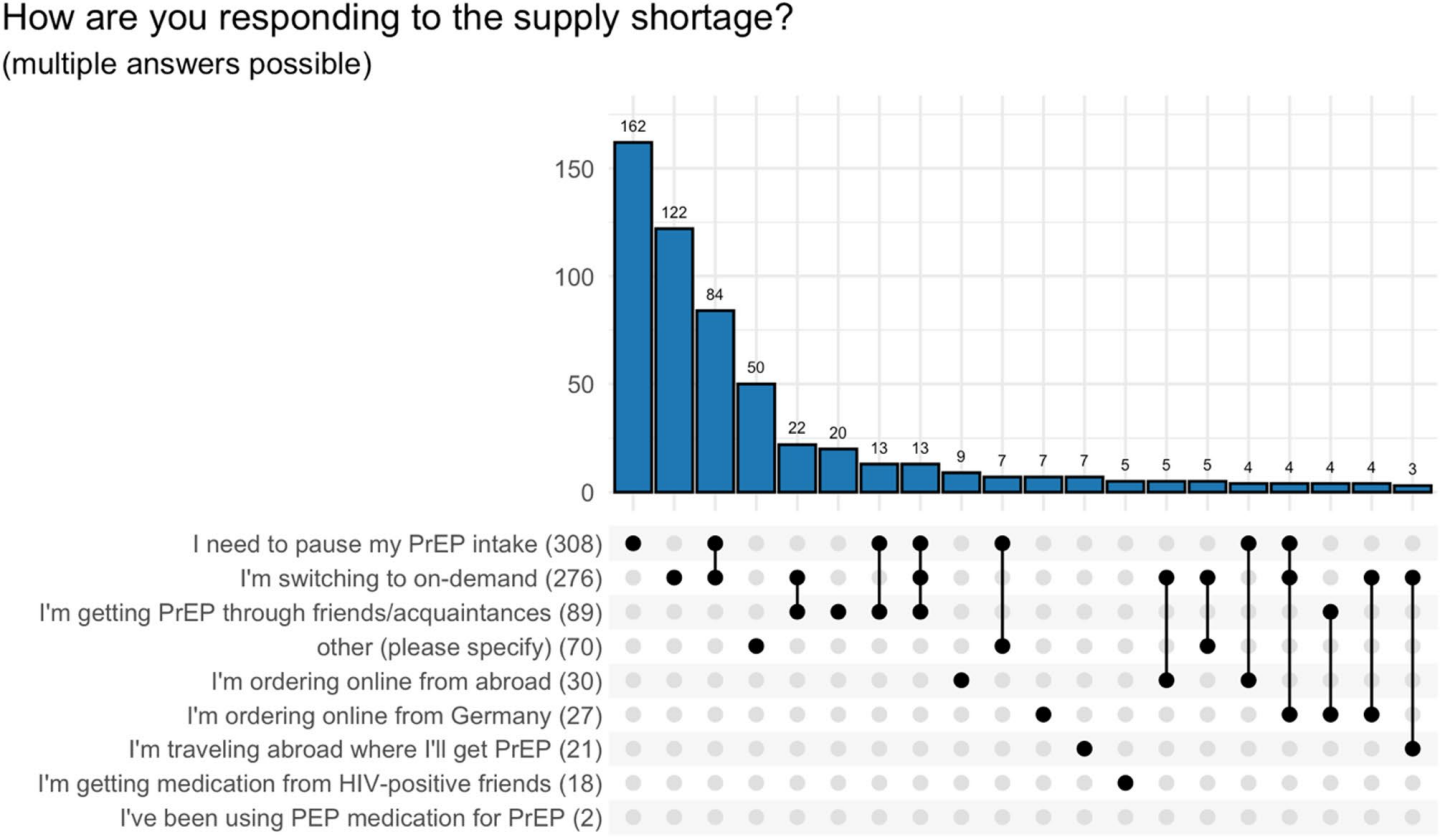
Reaction to the supply shortage of PrEP users who were affected by the supply shortage (*N* = 585), multiple answers possible (*N* = 841) (answer combinations of 35 participants with less than 3 occurrences were omitted)

PrEP users who were affected by the supply shortage (*N* = 585) predominantly stated that they had to pause PrEP (*N* = 308, 53%) or switch to taking it on-demand (*N* = 276, 47%) as a reaction to the supply shortage (multiple answers possible). In addition, 89 (15%) stated that they obtained PrEP from friends or acquaintances. Among the 70 participants who indicated ‘other’ strategies, frequently reported approaches included using existing stock or adjusting dosing; contacting multiple or specialized pharmacies, including time-consuming searches and incurring additional costs; using alternative procurement channels or medications; and making behavioral adjustments in dosing or sexual practices. PrEP users often used several strategies to deal with the supply shortage as indicated by the multiple responses (*N* = 833), see Fig. 5.

## Number of different sexual partners

The 585 PrEP users who were affected by the supply shortage were asked: “How many different partners have you had anal and/or vaginal intercourse with in the last six months?“. Most frequently more than 20 partners were stated (*N* = 154, 26%), followed by 6–10 partners (*N* = 137, 23%) (Table 1).

## HIV prevention behavior

**Fig. 6:**
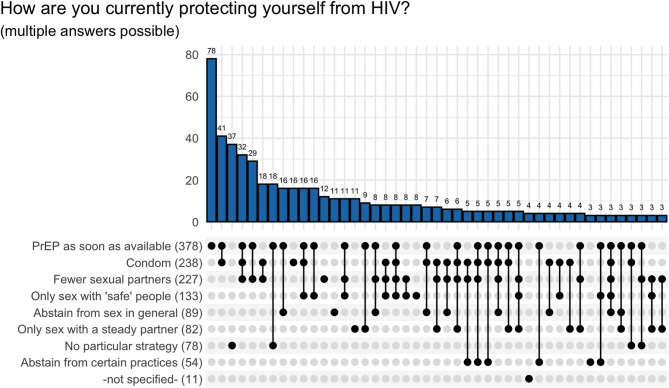
HIV prevention behavior of PrEP users who were affected by the supply shortage (*N* = 585), multiple answers possible (*N* = 1290) (answer combinations of 70 participants with less than 3 occurrences were omitted); Only sex with ‘safe’ people refers to sexual partners who are known to take PrEP, whose HIV viral load is below the detection limit, who have a negative HIV test

With regard to HIV prevention behavior, the 585 PrEP users who were affected by the supply shortage predominantly stated that they would stick to PrEP as the HIV prevention method as soon as it would be available again (*N* = 378, 65%), followed by condoms (*N* = 238, 41%), fewer sexual partners (*N* = 227, 39%) and limiting sexual contact to people who are assumed to pose no HIV risk, e.g. because they take PrEP, have a viral load below the detection limit or have a negative HIV test (*N* = 133, 23%). As in the case of PrEP supply sources PrEP users also often used several HIV protection strategies, see multiple responses (*N* = 1288) in Fig. 6.

## Perception of HIV protection

Around half (*N* = 299, 51%) of PrEP users who were affected by the supply shortage stated that they felt not protected or less well protected by their protection strategy, while the other half (*N* = 286, 49%) stated that they felt well or very well protected (Table 1).

## Perception of stress

**Fig. 7:**
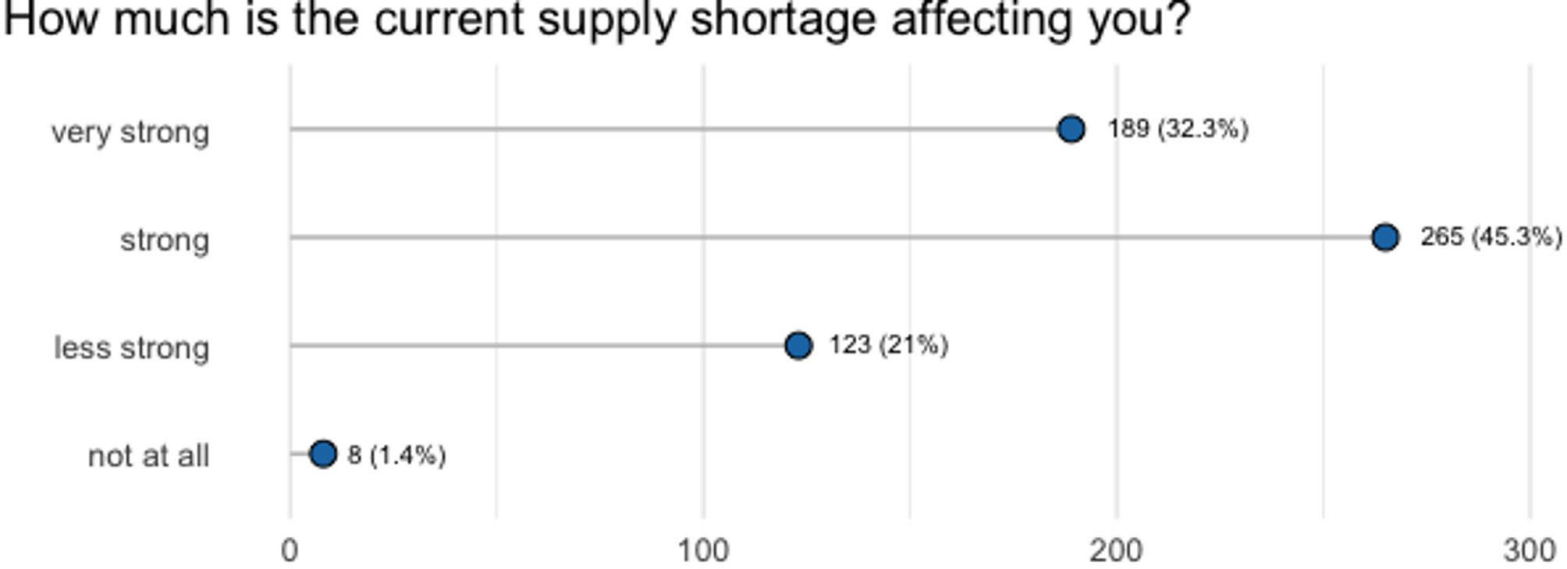
Perception of stress among PrEP users who were affected by the supply shortage (*N* = 585)

However, when asked about their perception of stress, the vast majority (*N* = 454, 78%) of PrEP users affected by the supply shortage reported high or very high levels of stress due to the situation of supply shortage (Fig. 7).

### Statistical analysis of factors associated with perception of stress

After adjusting for age, PrEP intake mode, perception of HIV protection, number of sexual partners in the past six months, and residential area, several significant associations with high or very high levels of stress were found. Perception of HIV protection was strongly associated with stress. Compared to those who felt very well protected, participants who felt less well protected had more than 5 times the odds of reporting high stress, and those who did not feel well protected had 19 times the odds. Participants using PrEP on-demand with less than 20 pills per month were significantly less likely to report high stress compared to those with daily PrEP use. More than 11 different sexual partners in the past six months was linked with significantly higher odds of perceived stress. Age and residential area were not significantly associated with perceived stress in the adjusted model (Table 2).

**Table 2:** Logistic regression with outcome perception of stress (high or very high levels of stress) adjusted for age, PrEP intake mode, perception of HIV protection, number of different sexual partners (anal, vaginal) in the last six months and residential area of participants by population size

		*N*	%	aOR	95% CI	95% CI	Pr(>|z|)	
Total	(Intercept)	570	100.0%	0.18	0.01	3.80	0.269	
Age	16–20	3	0.5%	Ref.				
	21–25	28	4.9%	1.61	0.10	26.68	0.740	
	26–30	64	11.2%	1.71	0.11	26.10	0.700	
	31–35	115	20.2%	2.78	0.19	41.13	0.457	
	36–40	100	17.5%	2.30	0.16	34.09	0.545	
	41–45	100	17.5%	2.97	0.20	44.10	0.428	
	46–50	67	11.8%	2.53	0.17	38.24	0.502	
	51–60	79	13.9%	2.23	0.15	33.14	0.560	
	> 60	14	2.5%	3.60	0.16	81.50	0.421	
PrEP use	daily	471	82.6%	Ref.				
	on-demand (> 20 pills/month)	51	8.9%	0.77	0.36	1.64	0.501	
	**on-demand (< 20 pills/month)**	**48**	**8.4%**	**0.33**	**0.16**	**0.65**	**0.001**	**
Perception of HIV protection	I feel very well protected	109	19.1%	Ref.				
	**I feel well protected**	**170**	**29.8%**	**1.76**	**1.02**	**3.03**	**0.043**	*
	**I feel less well protected**	**190**	**33.3%**	**5.39**	**2.91**	**9.96**	**< 0.001**	***
	**I don’t feel well protected**	**101**	**17.7%**	**19.24**	**6.32**	**58.62**	**< 0.001**	***
Number of different sexual partners (anal or vaginal) of PrEP users in the last six months	0	10	1.8%	Ref.				
	1	18	3.2%	1.57	0.26	9.61	0.626	
	2–3	77	13.5%	2.80	0.57	13.76	0.204	
	4–5	83	14.6%	2.37	0.49	11.57	0.286	
	**6–10**	**134**	**23.5%**	**4.06**	**0.85**	**19.35**	**0.078**	.
	**11–20**	**81**	**14.2%**	**5.05**	**1.01**	**25.23**	**0.048**	*
	**More than 20**	**152**	**26.7%**	**6.89**	**1.42**	**33.41**	**0.017**	*
	**I don’t know/don’t want to answer**	**15**	**2.6%**	**14.90**	**1.13**	**197.23**	**0.040**	*
Residential area of participants by population size	Small town or rural area (< 200,000 inhabitants)	191	33.5%	Ref.				
	Medium-sized city ( > = 200,000-<500,000 inhabitants)	68	11.9%	0.88	0.41	1.87	0.742	
	Large city ( > = 500,000 inhabitants)	311	54.6%	0.86	0.52	1.41	0.541	

Signif. codes: 0 ‘***’ 0.001 ‘**’ 0.01 ‘*’ 0.05 ‘.’ 0.1 ' ' 1

### Comparison of PrEP users and people with unmet PrEP demand

**Fig. 8:**
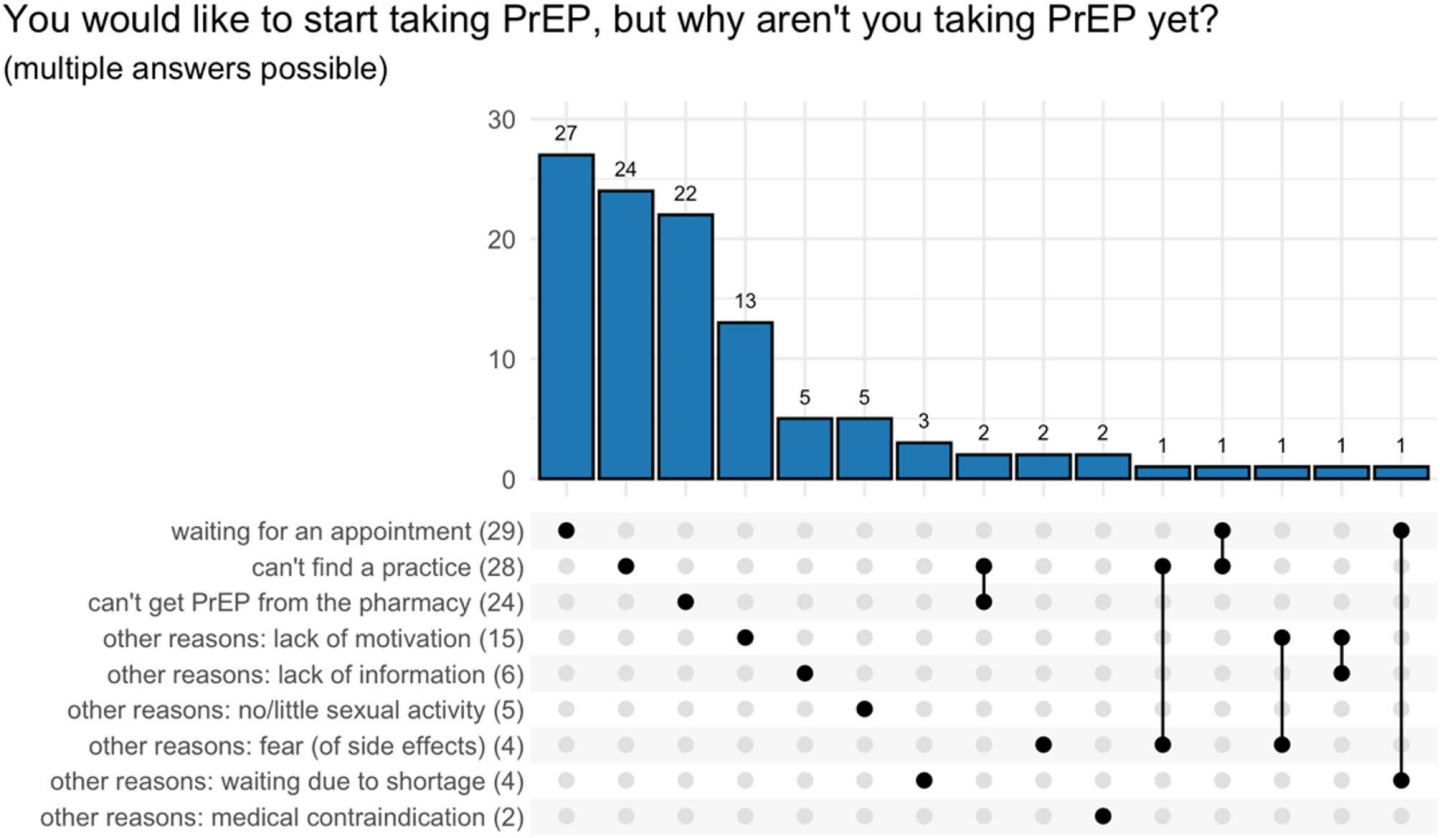
Reasons for unmet PrEP demand (*N* = 113), multiple answers possible (*N* = 117), inconsistent answers of 3 participants are not shown

We asked the participants: “Would you like to start PrEP?” With either Yes or No as an option. Participants who indicated Yes are referred to as people with unmet PrEP demand. The following were the main reasons given for not taking PrEP yet (multiple answers possible): I am waiting for an appointment to be prescribed PrEP (*N* = 29, 26%), I cannot find a doctor or medical center (*N* = 28, 25%), I have a PrEP prescription but cannot get PrEP from the pharmacy (*N* = 24, 21%), Lack of motivation / indecision (*N* = 15, 13%), see Fig. 8. Note that the ‘other reasons’ (given in free text form) of 37 participants were grouped by the authors.

A comparison of the characteristics of PrEP users and people with unmet PrEP demand revealed differences between the groups (Table 1). For example, the sex assigned at birth of PrEP users was 98% male, 2% female and 0.4% diverse. People with unmet PrEP demand, on the other hand, were significantly more likely to report that their sex assigned at birth was not male (chi²-Test(2) = 30.62, *p* < 0.001). People with unmet PrEP demand were 88% male, 9% female and 3.5% diverse (Table 1).

Similar results with a higher proportion of non-male participants were found in relation to the gender identities of PrEP users compared to people with unmet PrEP demand (Table 1). While 97% of PrEP users identified as men for people with unmet PrEP demand 87% identified as men.

There were also differences in the age distribution of the two groups. People with unmet PrEP demand were younger, 14% were in the 21–25 age group, whereas among PrEP users, 5% were in this age group, and the proportion of PrEP users was correspondingly higher in the older age groups (Table 1).

## Statistical analysis of PrEP users and people with unmet PrEP demand

Table 3 displays the results of a logistic regression model examining associations with unmet PrEP demand, adjusted for sex assigned at birth, age, and residential area by population size. Sex assigned at birth was significantly associated with unmet PrEP demand. Compared to individuals assigned male at birth, those assigned female and diverse had significantly higher odds. Living in a large city was associated with significantly lower odds of reporting unmet PrEP demand compared to those living in small towns or rural areas. Age was not significantly associated with unmet PrEP demand in the adjusted model (Table 3).

**Table 3:** Logistic regression with outcome unmet PrEP demand adjusted for sex assigned at birth, age and residential area of participants by population size

		*N*	%	aOR	95% CI	95% CI	*P*	
Total	(Intercept)	1044	100.0%	0.34	0.06	1.90	0.219	
Sex assigned at birth	Male	1005	96.3%	Ref.				
	**Female**	**29**	**2.8%**	**4.82**	**1.99**	**11.70**	**< 0.001**	***
	**Diverse**	**10**	**1.0%**	**7.91**	**1.81**	**34.50**	**0.006**	**
Age	16–20	8	0.8%	Ref.				
	21–25	68	6.5%	1.01	0.17	6.16	0.987	
	26–30	123	11.8%	0.55	0.09	3.34	0.520	
	31–35	197	18.9%	0.41	0.07	2.43	0.326	
	36–40	191	18.3%	0.46	0.08	2.71	0.391	
	41–45	168	16.1%	0.30	0.05	1.82	0.190	
	46–50	121	11.6%	0.38	0.06	2.39	0.304	
	51–60	138	13.2%	0.40	0.07	2.41	0.315	
	> 60	30	2.9%	0.27	0.03	2.52	0.249	
Residential area of participants by population size	Small town or rural area (< 200,000 inhabitants)	351	33.6%	Ref.				
	Medium-sized city ( > = 200,000-<500,000 inhabitants)	129	12.4%	0.66	0.34	1.29	0.225	
	**Large city** **( > = 500**,**000 inhabitants)**	**564**	**54.0%**	**0.56**	**0.36**	**0.88**	**0.012**	*

Signif. codes: 0 ‘***’ 0.001 ‘**’ 0.01 ‘*’ 0.05 ‘.’ 0.1 ' ' 1

## Discussion

In summary, we found that the majority of participants were affected by the TDF/FTC supply shortage. Further, the majority of PrEP users who were affected by the TDF/FTC shortage either had to pause PrEP or switch to PrEP on-demand. Perception of high or very high levels of stress was significantly more likely for daily PrEP use, lower perceived protection against HIV, and higher partner numbers with more than 11 different sexual partners in the last six months. People with unmet PrEP demand were significantly more likely female or diverse compared to male, although total number of non-male participants was small. We observed indications of gaps in PrEP coverage outside of large cities and a lack of availability of medical facilities that prescribe PrEP. For people with unmet PrEP demand who experience barriers in motivation and indecisiveness, targeted and individual PrEP counseling could help to determine the actual need and clarify the personal benefits of PrEP.

### Reaction to the supply shortage

The majority of PrEP users who were affected by the TDF/FTC shortage either had to pause PrEP or switch to taking it on-demand. This is worrying, as the results of the EvE-PrEP and PrEP-Surv projects in Germany showed that the few HIV infections associated with PrEP use primarily occurred during periods of PrEP interruption or irregular on-demand use [10]. As the adjustment and change of PrEP intake was not a voluntary decision but due to the shortage, it might be more difficult for those affected to adapt their sexual behavior to the new situation. This harbors the risk of irregular intake or PrEP interruption despite ongoing HIV risks. It would be beneficial to monitor this development and to investigate HIV infections related to supply shortage in future surveys and studies.

### Reaction to the supply shortage, HIV prevention behavior and perception of stress

Most PrEP users had several strategies to either respond to the supply shortage or to protect themselves against HIV. About two third of PrEP users stated to stick to PrEP as an HIV prevention method as soon as it would be available again. Followed by condoms and fewer sexual partners which was reported by about 40%, respectively and about one fifth that reported limiting sexual contact to people who are assumed to pose no HIV risk. PrEP was therefore the most preferred HIV prevention method. The overall perception of protection was relatively balanced, between a good and less good perception of protection. However, PrEP users who were affected by the supply shortage predominantly reported high or very high levels of stress. The adjusted analysis of the perception of stress and various factors showed that PrEP users with high or very high levels of stress were significantly more likely to feel not well or less well protected against HIV. In addition, they were significantly more likely to report having more than 11 sexual partners and significantly less likely to report taking PrEP with less than 20 pills per month. It seems plausible that a lower perception of HIV protection contributes to increased stress. It also seems plausible that a higher number of sexual partners and daily PrEP is perceived as stressful in a situation with an unsecured supply of medication.

The number of different sexual partners in the last six months showed that PrEP is mostly used by people with a higher number of sexual partners, which is in line with the indication for PrEP use.

With regard to the first start of PrEP, a decline in PrEP starts was observed during the COVID-19 pandemic in 2020 and 2021. Effects of the COVID-19 pandemic on the number of PrEP users, PrEP starts and health seeking behavior has also been observed in other data sources [11–15]. Although the number of PrEP starts rose again afterwards, it remains uncertain how PrEP would have developed without the pandemic. The after-effects of the pandemic, particularly with regard to barriers to accessing PrEP, are likely still to be felt for some time to come. In addition, other health threats such as the Mpox virus outbreak 2022 also influenced the sexual behavior and healthcare seeking behavior of MSM [16], who make up the majority of PrEP users, and might have led to reduced sexual activity and PrEP use during the outbreak.

### PrEP user characteristics and comparison of PrEP users and people with unmet PrEP demand

The majority of PrEP users in our survey were male. The proportion of 98% male PrEP users is in line with what has been observed in previous surveys and health insurance data in Germany [3, 17]. In some studies in countries where PrEP availability or coverage has been established for a longer period of time like France and the United States (US) slightly higher proportions of female PrEP users of around 2.5% and 4.9% were seen [15, 18]. Although in the US study, women were also less likely to receive a PrEP prescription and initiate PrEP and more likely to discontinue PrEP [18]. A discrepancy between a PrEP need and receiving a PrEP prescription, as well as a shorter duration of PrEP use, has also been observed in Germany [19, 20]. Australia, where PrEP has been government subsidized since April 2018 shows a similar proportion of 98% men [21]. The adjusted analysis of the characteristics of PrEP users and people with unmet PrEP demand in our survey showed that people with unmet PrEP demand were significantly more likely female or diverse compared to male, though numbers of non-male participants were small. PrEP users also more often identified as men compared to people with unmet PrEP demand. In addition, the proportion of people with unmet PrEP demand was significantly lower in large cities, which indicates gaps in coverage outside of large cities. The barriers and gaps in PrEP provision observed in our survey are consistent with the information provided by the Community Advisory Board and other analyses within the PrEP surveillance project which have revealed access barriers to PrEP among non-male individuals and gaps in provision outside of metropolitan areas in Germany [3, 22]. A lack of PrEP provision for women and barriers to PrEP uptake were also observed in a systematic literature review on PrEP for women in Europe. The authors further state that barriers to PrEP uptake are complex and rooted in institutional and societal stigma, which must be addressed at policy level [23]. These conclusions are in line with our findings and suggestions.

Other major barriers to accessing PrEP mentioned by people with unmet PrEP demand were the lack of availability of appointments at medical facilities that prescribe PrEP and the lack of PrEP drugs. The need for more facilities authorized to prescribe PrEP, as well as for solutions to capacity bottlenecks and the overburdening of existing care structures, has already been identified in several surveys conducted as part of the EvE-PrEP and PrEP-Surv, as well as in discussions with the PrEP Surveillance Community Advisory Board [24]. There is an urgent need for further concepts to ensure the long-term provision of PrEP in Germany. Practical proposals for this were developed by the PrEP-Surv Community Advisory Board and published in a statement and position paper [25].

On the one hand, there is an urgent need for education and information beyond the group of men, who have sex with men (MSM). This includes people in sex work, people who use drugs, people from countries with a higher HIV prevalence, trans* and non-binary people, and generally all people with frequently changing sexual partners and an increased risk of HIV. On the other hand, it is important to remove structural barriers to PrEP access, such as access for people without health insurance or a valid residence permit and the lack of availability of PrEP prescribers, especially outside of large cities. The existing supply structures in Germany are already at the limits of their capacity and do not adequately reach certain groups in need of PrEP. Other specialties from the fields of gynecology, general and travel medicine and psychiatry could and should be much more involved in PrEP provision. In addition, existing community structures such as checkpoints could be used and PrEP in the public health service could be expanded even further. The expansion and use of telemedicine, including e-prescription and home testing, could also help to make PrEP more widely available. In its July 2022 update the WHO implementation guidance emphasized the importance of differentiated, simplified, demedicalized and comprehensive PrEP services in order to support PrEP uptake, persistence and effective use and assist efforts to achieve global goals set out in the 2022–2030 Global Health Sector Strategies on HIV, viral hepatitis and sexually transmitted infections [26]. Several other studies have also pointed out the need for a broader access to PrEP that includes women and different concepts of PrEP provision [23, 27–29].

In addition, 12% of people with unmet PrEP demand stated that they experienced a lack of motivation or indecision as barriers. In these cases, targeted, individual PrEP counseling could help to determine the actual need and clarify the personal benefits of PrEP. The importance of adequate counseling was also pointed out in the WHO implementation guidance update [26].

## Limitations

The present study has several limitations that should be considered when interpreting the results. Due to the anonymous online survey design, it cannot be ruled out that individual participants may have completed the survey more than once. However, from the authors’ perspective, the likelihood appears low, as there is no evident rationale or benefit associated with doing so. Verification of the information provided was not possible, which means that potential false statements or misunderstandings may have gone unnoticed. There is also a potential for self-selection bias due to the use of a convenience sample as well as social desirability bias. It is likely that individuals with personal experiences related to supply shortages or with an unmet PrEP demand were particularly motivated to participate. This limits the generalizability of the findings to the overall population of PrEP users. However, the survey was explicitly aimed at these specific populations, so the focus appears methodologically justified. In addition, the sample is limited to individuals with internet access and the corresponding digital literacy. Population groups with limited access to digital media, such as older adults or socioeconomically disadvantaged individuals, may therefore be systematically underrepresented. However, the impact is considered to be rather low. The likelihood of recall bias is considered low in this case, as the survey period immediately followed the supply shortages, meaning that relevant events were still recent. Given the cross-sectional design, odds ratios for relatively common outcomes may overestimate the corresponding prevalence ratios and thus exaggerate the magnitude of the association. Further, due to the cross-sectional design, the temporal sequence between exposures and outcomes cannot be established; therefore, observed associations should not be interpreted as causal. Overall, the results should be interpreted with caution in light of these limitations. They primarily provide insights for the before mentioned groups.

## Conclusions

The majority of participants were affected by the TDF/FTC supply shortage. Further, we think it is concerning that the majority of PrEP users who were affected by the TDF/FTC shortage were forced to either pause PrEP or switch to on-demand use. Since these changes in intake were not voluntary, they may have hindered users’ ability to adapt their sexual behavior accordingly, increasing the risk of irregular PrEP use or interruptions despite ongoing HIV exposure. Further research is needed to investigate whether HIV infections occurred as a consequence of the supply shortage.

PrEP users most at risk and most reliant on PrEP for protection experience greater psychological burden during supply disruptions. Healthcare systems should prioritize consistent and uninterrupted access to PrEP for those individuals.

People with unmet PrEP demand were significantly more likely female or diverse compared to male, although total numbers of non-male participants were small. PrEP users more often identified as men compared to people with unmet PrEP demand. Moreover, indications of gaps in PrEP coverage outside of large cities and a lack of availability of medical facilities that prescribe PrEP were observed. There is an urgent need to expand education and outreach beyond the population of MSM. Increasing the number of PrEP prescribers and developing sustainable strategies to secure long-term access to PrEP in Germany are essential steps. Practical solutions for these challenges have already been developed and published within the PrEP Surveillance project and can serve as a foundation for further implementation.

Targeted, individual PrEP counseling could help to determine the actual need and clarify the personal benefits of PrEP for those with barriers in motivation and indecisiveness.

## Supplementary Information

Below is the link to the electronic supplementary material.

Supplementary Material 1

## Data Availability

The datasets used and/or analysed during the current study are available from the corresponding author on reasonable request.

## Abbreviations

BMG: German Federal Ministry of Health
EvE-PrEP: PrEP evaluation project
Dagnä: German Association of Outpatient Physicians for Infectious Diseases and HIV Medicine
DAIG: German AIDS Society
DSTIG: German STI Society
MSM: Men, who have sex with men
PrEP: HIV pre-exposure prophylaxis
PrEP-Surv: Surveillance of HIV PrEP within the statutory health insurance system in Germany
RKI: Robert Koch Institute
SHI: Statutory health insurance
TDF/FTC: Tenofovir disoproxil/emtricitabine
