# Supplementary material for: Unsuspected drug shortage is impacting the German HIV PrEP supply - results of a cross-sectional survey show: majority of PrEP users had to stop or switch to on-demand PrEP and higher unmet PrEP demand among women, diverse individuals, and those in rural or small-town areas
Source: BMC Infect Dis. 2025 Nov 13;25:1574. doi: 10.1186/s12879-025-12086-9 (PMC12616934; doi:10.1186/s12879-025-12086-9)
Supplement: Supplementary file 1 — Supplementary Material 1 [file 12879_2025_12086_MOESM1_ESM.pdf]

INTRO_PAGE
There are currently huge supply shortages of HIV PrEP in Germany.

If you use PrEP or have ever thought about it, we would like to invite you to take part in a short online survey. The survey is being conducted by the Robert Koch Institute as part of the PrEP Surveillance project.

The aim is to better understand the impact on PrEP users and those interested in PrEP. This is important for future PrEP provision.

The survey is short, approximately 10 questions with an estimated completion time of maximal of 5 minutes.

Your participation is voluntary, anonymous and cannot be traced. Please give honest and, if possible, complete answers. These are very valuable for a comprehensive picture of the current situation and the future of PrEP!

We thank you in advance for your valuable contribution. The PrEP-Surv Team

Would you like to start the survey now?

- ○ Yes
- ○ No

ALTER
How old are you?

Select an answer...

GESCHLECHT
Which gender was assigned to you at birth (entry in birth certificate)?

- ○ Male
- ○ Female
- ○ Divers

GESCHLECHT_IDENT
Which gender do you currently identify with? (multiple answers possible)

- ☐ Male
- ☐ Female
- ☐ Trans-Man
- ☐ Trans-Woman
- ☐ Non binary
- ☐ Inter
- ☐ Agender/No gender
- ☐ Genderfluid
- ☐ No answer

PLZ
Where do you live? Please enter the first 3 postal code digits.

- No answer
- Refusal
- Do not know

PREP_NUTZENDE
Have you taken HIV-PrEP in the last 6 months?

- Yes
- No

PREP_INTEREST
Would you like to start PrEP?

- Yes
- No

PREP_INTEREST2
You would like to start PrEP, why are you not currently taking PrEP? (multiple answers possible)

- I can't find a doctor or center
- I am waiting for an appointment to get PrEP prescription
- I have a PrEP prescription, but can't get PrEP at the pharmacy
- Other reason (please specify)

EINNAHME_SEIT

When did you start PrEP for the first time?

	Month	Year
	Select an answer...	Select an answer...

EINNAHME_MODUS
How do you usually take PrEP?

- Continuously/daily
- Event driven/on-demand more than 2/3 of the time (≥20 tablets per month)
- Event driven/on-demand less than 2/3 of the time (<20 tablets per month)

LIEFERENG_BETROFFEN
Are you affected by the current shortages, meaning have you already experienced difficulties getting PrEP in the current situation?

- ○ Yes
- ○ No

LIEFERENG_NEIN
Where do you currently getting PrEP from? (multiple answers possible)

- ☐ I get PrEP from the doctor and pharmacy as usual
- ☐ I currently take it daily and still have PrEP
- ☐ I am currently taking event driven/on-demand and still have PrEP
- ☐ I get PrEP through friends/acquaintances
- ☐ I order on the Internet/online from Germany
- ☐ I order on the Internet/online from abroad
- ☐ I travel abroad, where I get PrEP
- ☐ I have used PEP medication for PrEP
- ☐ I have received medication from HIV positive friends/acquaintances
- ☐ Other (please specify)

LIEFERENG_SEIT

Since when have you been affected by the shortages?

	Month	Year
**since**	Select an answer...	Select an answer...

REAG
How do you react to the shortages? (multiple answers possible)

- ☐ I have to pause/stop taking PrEP
- ☐ Switch to event driven/on-demand so that I can take my PrEP for longer
- ☐ I get PrEP through friends/acquaintances
- ☐ I order on the Internet/online from Germany
- ☐ I order on the Internet/online from abroad
- ☐ I travel abroad, where I get PrEP
- ☐ I have used PEP medication for PrEP
- ☐ I have received medication from HIV positive friends/acquaintances
- ☐ Other (please specify)

SCHUETZ
How do you currently protect yourself against HIV? (multiple answers possible)

- ☐ Condom
- ☐ Fewer sexual partners
- ☐ Only sex with a steady partner
- ☐ Abstinence from sex in general
- ☐ Abstaining from certain practices (anal intercourse, chemsex)
- ☐ Only have sex with people who I know are taking PrEP, whose viral load is below the detection limit, who have a negative HIV test
- ☐ PrEP if available
- ☐ No special strategy
- ☐ Other protection (please specify)

SCHUETZ_2
How protected do you feel at the moment with your protection strategy?

- ○ I feel very well protected
- ○ I feel well protected
- ○ I feel less well protected
- ○ I do not feel well protected

N_SEXPARTNER
With how many different partners have you had anal and/or vaginal intercourse in the last 6 months?

- 0
- 1
- 2-3
- 4-5
- 6-10
- 11-20
- More than 20
- I don't know/don't want to answer

BELASTUNG
How much of a burden is the current situation of shortages to you?

- Very much
- Much
- Less much
- Not at all

INT99

You decided not to participate. Click on "Next" to complete the survey.

INT97

Thank you for your answers! Can we ask you one more thing? There is currently another large survey of men who have sex with men: EMIS survey
Motto: EMIS: Your voice - our strength

Your answers will help to promote the interests of gay and bisexual men. We ask you to fill in this questionnaire too! Click here: EMIS survey
Please click on finish to complete the survey.
